# Dibenzo-18-crown-6

**DOI:** 10.1107/S1600536808030250

**Published:** 2008-09-24

**Authors:** Geraldo M. de Lima, James L. Wardell, William T. A. Harrison

**Affiliations:** a Departamento de Quimica, Universidade Federal de Minas Gerais, UFMG, Avenida Antônio Carlos 6627, Belo Horizonte, MG, CEP 31270-901, Brazil; b Department of Chemistry, University of Aberdeen, Meston Walk, Aberdeen AB24 3UE, Scotland

## Abstract

The asymmetric unit of the title compound, C_20_H_24_O_6_, contains two mol­ecules that are identical within standard deviations concerning bond lengths and angles as well as their conformations. In the crystal structure, weak C—H⋯O inter­actions help to consolidate the packing.

## Related literature

For background, see: Hutton & Oakes (1976 ▶); Baur & Kassner (1992 ▶); Grotjahn *et al.* (2001 ▶); Barranikov *et al.* (2002 ▶); Su *et al.* (2003 ▶). For bond-length data, see: Allen *et al.* (1987 ▶).
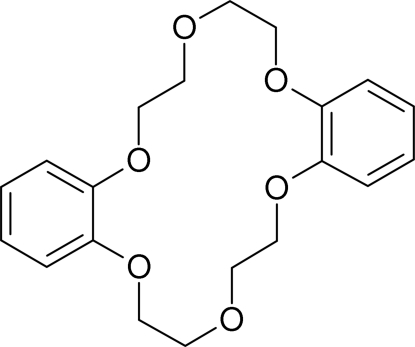


## Experimental

### 

#### Crystal data

C_20_H_24_O_6_
*M* *_r_* = 360.39
Monoclinic, 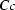

*a* = 4.902 (3) Å
*b* = 28.58 (2) Å
*c* = 25.06 (2) Å
β = 92.049 (8)°
*V* = 3509 (4) Å^3^
*Z* = 8
Synchrotron radiation
λ = 0.6946 Å
μ = 0.10 mm^−1^
*T* = 120 (2) K
0.04 × 0.02 × 0.02 mm

#### Data collection

Bruker SMART APEXII CCD diffractometer
Absorption correction: none
13644 measured reflections
3564 independent reflections
2972 reflections with *I* > 2σ(*I*)
*R*_int_ = 0.054

#### Refinement

*R*[*F* ^2^ > 2σ(*F* ^2^)] = 0.098
*wR*(*F* ^2^) = 0.294
*S* = 1.15
3564 reflections
470 parameters
2 restraints
H-atom parameters constrained
Δρ_max_ = 0.50 e Å^−3^
Δρ_min_ = −0.52 e Å^−3^

### 

Data collection: *APEX2* (Bruker, 2004 ▶); cell refinement: *SAINT* (Bruker, 2004 ▶); data reduction: *SAINT*; program(s) used to solve structure: *SHELXS97* (Sheldrick, 2008 ▶); program(s) used to refine structure: *SHELXL97* (Sheldrick, 2008 ▶); molecular graphics: *ORTEP-3* (Farrugia, 1997 ▶); software used to prepare material for publication: *SHELXL97*.

## Supplementary Material

Crystal structure: contains datablocks I, global. DOI: 10.1107/S1600536808030250/im2081sup1.cif

Structure factors: contains datablocks I. DOI: 10.1107/S1600536808030250/im2081Isup2.hkl

Additional supplementary materials:  crystallographic information; 3D view; checkCIF report

## Figures and Tables

**Table 1:** Hydrogen-bond geometry (Å, °)

*D*—H⋯*A*	*D*—H	H⋯*A*	*D*⋯*A*	*D*—H⋯*A*
C16—H16⋯O8^i^	0.95	2.57	3.46 (1)	157
C26—H26⋯O5^ii^	0.95	2.54	3.42 (1)	155

Symmetry codes: (i) 
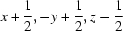
; (ii) 
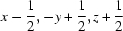
.

**Fig. 1.**
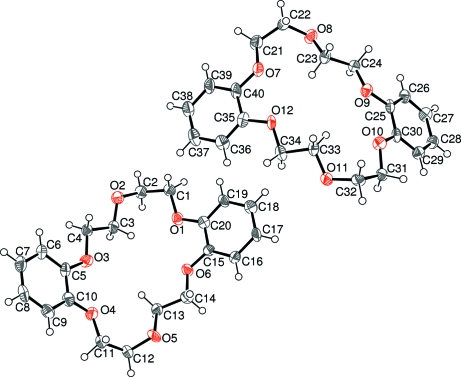
View of the molecular structure of (I) showing 50% displacement ellipsoids. The H atoms are drawn as spheres of arbitrary radius.

**Fig. 2.**
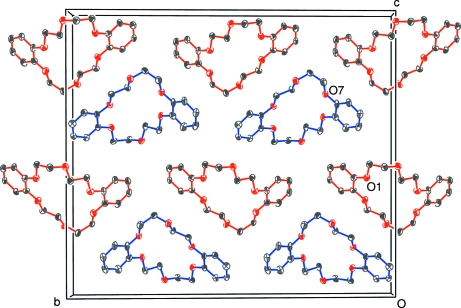
The packing in (I), viewed down [100] with H atoms omitted for clarity. Bonds in the C1 and C21 molecules are coloured red and blue, respectively.

## supplementary crystallographic information

### Comment

The relationship between the conformations of crown ethers and their coordinating abilities has been the subject of many crystallographic, spectroscopic, thermochemical and theoretical studies (*e.g.* Grotjahn *et al.*, 2001; Barranikov *et al.*, 2002; Su *et al.*, 2003). The crystal structures of a large number of complexes containing the title crown ether have been determined, but the structure of the title compound, (I), the free crown ether, has remained undetermined until now.

There are two molecules in the asymmetric unit of (I) (Fig. 1) with very similar conformations, with both possessing local approximate C_2_ symmetry. This is also reflected in the *trans*—gauche—trans—gauche sequence of conformation angles about the four O—C—C—O bonds in the 18-membered ring. The dihedral angles between the mean planes of the aromatic rings are 65.5 (3)° for the C1 molecule and 66.1 (3)° for the C21 molecule. Otherwise, the geometrical paramaters for (I) may be regarded as normal (Allen *et al.*, 1987).

In the crystal of (I), two weak intermolecular C—H···O interactions (Table 1) may help to consolidate the packing. There are no π-π stacking interactions in (I), the minimum ring centroid separation being greater than 5.6 Å. The packing (Fig. 3) for (I) results in (001) pseudo layers of the two asymmetric molecules.

### Experimental

The unreacted title compound was obtained from the attempted complexation with 3-(trichlorostannyl)propanamide (Hutton & Oakes, 1976). Slow evaporation of dibenzo-18-crown-6 and 3-(trichlorostannyl)propanamide (0.2 mmol of each) in ethanol solution (15 ml) led to isolation of tiny colourless shards of (I).

### Refinement

The situation of two asymmetric molecules in space group *Cc* is a suspicious one (Baur & Kassner, 1992) and careful checks for additional or missed crystal symmetry were made, but none was found. No starting models could be established in space groups *C*2/*c*, C2/m or any lower symmetry centrosymmetric space groups. Structure solutions in lower-symmetry non-centrosymmetric space groups were easily achieved and could all be transformed to the model described above.

Even with the use of synchrotron radiation, the small crystal size resulted in weak diffraction and a poor data to parameter ratio of 7.6:1. The residuals are also high. Anomalous dispersion was negligible and Friedel pairs were merged before refinement. The hydrogen atoms were placed in calculated positions (C—H = 0.95–0.99 Å) and refined as riding with *U*_iso_(H) = 1.2*U*_eq_(C).

### Crystal data

**Table:**

C_20_H_24_O_6_	*F*(000) = 1536
*M**_r_* = 360.39	*D*_x_ = 1.365 Mg m^−^^3^
Monoclinic, *C**c*	Synchrotron radiation, λ = 0.6946 Å
Hall symbol: C -2yc	Cell parameters from 982 reflections
*a* = 4.902 (3) Å	θ = 2.9–25.3°
*b* = 28.58 (2) Å	µ = 0.10 mm^−^^1^
*c* = 25.06 (2) Å	*T* = 120 K
β = 92.049 (8)°	Shard, colourless
*V* = 3509 (4) Å^3^	0.04 × 0.02 × 0.02 mm
*Z* = 8	

### Data collection

**Table:**

Bruker SMART APEX2 CCD diffractometer	2972 reflections with *I* > 2σ(*I*)
Radiation source: Daresbury SRS station 9.8	*R*_int_ = 0.054
silicon 111	θ_max_ = 25.8°, θ_min_ = 2.9°
Fine–slice ω scans	*h* = −6→6
13644 measured reflections	*k* = −35→35
3564 independent reflections	*l* = −30→31

### Refinement

**Table:**

Refinement on *F*^2^	Secondary atom site location: difference Fourier map
Least-squares matrix: full	Hydrogen site location: inferred from neighbouring sites
*R*[*F*^2^ > 2σ(*F*^2^)] = 0.098	H-atom parameters constrained
*wR*(*F*^2^) = 0.294	*w* = 1/[σ^2^(*F*_o_^2^) + (0.1469*P*)^2^ + 19.0455*P*] where *P* = (*F*_o_^2^ + 2*F*_c_^2^)/3
*S* = 1.15	(Δ/σ)_max_ < 0.001
3564 reflections	Δρ_max_ = 0.50 e Å^−^^3^
470 parameters	Δρ_min_ = −0.52 e Å^−^^3^
2 restraints	Extinction correction: SHELXL97 (Sheldrick, 2008), Fc^*^=kFc[1+0.001xFc^2^λ^3^/sin(2θ)]^-1/4^
Primary atom site location: structure-invariant direct methods	Extinction coefficient: 0.067 (7)

### Special details

**Table:**

Geometry. All e.s.d.'s (except the e.s.d. in the dihedral angle between two l.s. planes) are estimated using the full covariance matrix. The cell e.s.d.'s are taken into account individually in the estimation of e.s.d.'s in distances, angles and torsion angles; correlations between e.s.d.'s in cell parameters are only used when they are defined by crystal symmetry. An approximate (isotropic) treatment of cell e.s.d.'s is used for estimating e.s.d.'s involving l.s. planes.
Refinement. Refinement of *F*^2^ against ALL reflections. The weighted *R*-factor *wR* and goodness of fit *S* are based on *F*^2^, conventional *R*-factors *R* are based on *F*, with *F* set to zero for negative *F*^2^. The threshold expression of *F*^2^ > σ(*F*^2^) is used only for calculating *R*-factors(gt) *etc*. and is not relevant to the choice of reflections for refinement. *R*-factors based on *F*^2^ are statistically about twice as large as those based on *F*, and *R*- factors based on ALL data will be even larger.

### Fractional atomic coordinates and isotropic or equivalent isotropic displacement parameters (Å^2^)

**Table:**

	*x*	*y*	*z*	*U*_iso_*/*U*_eq_	
C1	−0.212 (2)	0.0826 (4)	0.4641 (4)	0.033 (2)	
H1A	−0.3259	0.1107	0.4695	0.039*	
H1B	−0.0944	0.0776	0.4965	0.039*	
C2	−0.394 (2)	0.0398 (4)	0.4538 (4)	0.036 (2)	
H2A	−0.5394	0.0393	0.4802	0.043*	
H2B	−0.4828	0.0424	0.4178	0.043*	
C3	−0.127 (2)	−0.0154 (3)	0.4094 (4)	0.035 (2)	
H3A	0.0010	0.0093	0.3982	0.042*	
H3B	−0.2696	−0.0194	0.3807	0.042*	
C4	0.0261 (18)	−0.0614 (3)	0.4193 (4)	0.0295 (19)	
H4A	0.1473	−0.0591	0.4516	0.035*	
H4B	−0.1041	−0.0875	0.4241	0.035*	
C5	0.3565 (19)	−0.1064 (3)	0.3725 (4)	0.032 (2)	
C6	0.3652 (19)	−0.1415 (3)	0.4119 (4)	0.033 (2)	
H6	0.2444	−0.1407	0.4407	0.040*	
C7	0.555 (2)	−0.1773 (3)	0.4076 (4)	0.040 (2)	
H7	0.5648	−0.2013	0.4339	0.048*	
C8	0.728 (2)	−0.1785 (3)	0.3663 (5)	0.043 (3)	
H8	0.8561	−0.2033	0.3640	0.051*	
C9	0.719 (2)	−0.1436 (4)	0.3272 (4)	0.038 (2)	
H9	0.8412	−0.1447	0.2986	0.046*	
C10	0.532 (2)	−0.1076 (3)	0.3302 (4)	0.035 (2)	
C11	0.707 (2)	−0.0650 (3)	0.2567 (4)	0.032 (2)	
H11A	0.7199	−0.0925	0.2329	0.039*	
H11B	0.8852	−0.0604	0.2756	0.039*	
C12	0.630 (2)	−0.0222 (3)	0.2247 (4)	0.041 (2)	
H12A	0.7406	−0.0215	0.1924	0.049*	
H12B	0.4361	−0.0248	0.2128	0.049*	
C13	0.427 (2)	0.0340 (3)	0.2823 (4)	0.033 (2)	
H13A	0.3957	0.0113	0.3113	0.040*	
H13B	0.2627	0.0353	0.2584	0.040*	
C14	0.4934 (19)	0.0814 (4)	0.3048 (4)	0.033 (2)	
H14A	0.6710	0.0811	0.3249	0.039*	
H14B	0.5002	0.1051	0.2761	0.039*	
C15	0.2997 (17)	0.1272 (3)	0.3756 (4)	0.0277 (18)	
C16	0.4900 (18)	0.1624 (3)	0.3713 (4)	0.0294 (19)	
H16	0.6100	0.1628	0.3424	0.035*	
C17	0.505 (2)	0.1984 (3)	0.4110 (4)	0.038 (2)	
H17	0.6328	0.2231	0.4082	0.045*	
C18	0.3359 (19)	0.1972 (3)	0.4526 (4)	0.035 (2)	
H18	0.3461	0.2211	0.4790	0.042*	
C19	0.144 (2)	0.1603 (3)	0.4566 (4)	0.032 (2)	
H19	0.0274	0.1591	0.4861	0.038*	
C20	0.1258 (18)	0.1265 (3)	0.4179 (4)	0.031 (2)	
O1	−0.0471 (13)	0.0889 (2)	0.4184 (3)	0.0334 (15)	
O2	−0.2456 (14)	−0.0028 (2)	0.4572 (3)	0.0379 (16)	
O3	0.1848 (13)	−0.0692 (2)	0.3723 (3)	0.0350 (16)	
O4	0.4961 (14)	−0.0723 (2)	0.2945 (3)	0.0381 (17)	
O5	0.6677 (15)	0.0207 (3)	0.2526 (3)	0.0414 (18)	
O6	0.2725 (13)	0.0911 (2)	0.3399 (3)	0.0325 (15)	
C21	0.274 (2)	0.2188 (4)	0.7537 (5)	0.041 (2)	
H21A	0.2570	0.1907	0.7765	0.049*	
H21B	0.0967	0.2245	0.7348	0.049*	
C22	0.354 (2)	0.2602 (3)	0.7869 (4)	0.035 (2)	
H22A	0.2364	0.2614	0.8182	0.043*	
H22B	0.5448	0.2559	0.8005	0.043*	
C23	0.5683 (18)	0.3155 (3)	0.7319 (4)	0.0294 (19)	
H23A	0.5979	0.2925	0.7031	0.035*	
H23B	0.7303	0.3153	0.7566	0.035*	
C24	0.526 (2)	0.3640 (3)	0.7083 (4)	0.032 (2)	
H24A	0.3490	0.3656	0.6881	0.038*	
H24B	0.5258	0.3877	0.7371	0.038*	
C25	0.724 (2)	0.4109 (3)	0.6399 (4)	0.031 (2)	
C26	0.5268 (19)	0.4460 (3)	0.6442 (4)	0.032 (2)	
H26	0.4039	0.4457	0.6727	0.038*	
C27	0.5145 (17)	0.4811 (4)	0.6062 (4)	0.032 (2)	
H27	0.3801	0.5049	0.6082	0.039*	
C28	0.6990 (19)	0.4818 (3)	0.5647 (4)	0.032 (2)	
H28	0.6901	0.5065	0.5394	0.038*	
C29	0.8951 (18)	0.4469 (3)	0.5600 (4)	0.0307 (19)	
H29	1.0202	0.4478	0.5319	0.037*	
C30	0.9041 (18)	0.4109 (3)	0.5970 (4)	0.0283 (19)	
C31	1.2366 (19)	0.3687 (3)	0.5498 (4)	0.031 (2)	
H31A	1.3639	0.3953	0.5465	0.037*	
H31B	1.1140	0.3678	0.5176	0.037*	
C32	1.3939 (19)	0.3235 (3)	0.5552 (4)	0.033 (2)	
H32A	1.5297	0.3221	0.5269	0.040*	
H32B	1.4948	0.3232	0.5901	0.040*	
C33	1.1119 (19)	0.2686 (3)	0.6009 (4)	0.032 (2)	
H33A	0.9953	0.2936	0.6152	0.038*	
H33B	1.2594	0.2614	0.6277	0.038*	
C34	0.9432 (19)	0.2249 (3)	0.5878 (4)	0.034 (2)	
H34A	0.8174	0.2302	0.5566	0.041*	
H34B	1.0624	0.1979	0.5803	0.041*	
C35	0.625 (2)	0.1796 (4)	0.6351 (4)	0.037 (2)	
C36	0.604 (2)	0.1462 (4)	0.5958 (5)	0.038 (2)	
H36	0.7148	0.1484	0.5657	0.046*	
C37	0.420 (2)	0.1088 (4)	0.5998 (5)	0.044 (3)	
H37	0.4148	0.0850	0.5733	0.053*	
C38	0.246 (2)	0.1064 (4)	0.6417 (5)	0.043 (3)	
H38	0.1158	0.0819	0.6436	0.051*	
C39	0.266 (2)	0.1413 (3)	0.6819 (5)	0.039 (2)	
H39	0.1482	0.1401	0.7113	0.046*	
C40	0.453 (2)	0.1766 (3)	0.6787 (4)	0.037 (2)	
O7	0.4850 (14)	0.2117 (2)	0.7157 (3)	0.0348 (15)	
O8	0.3343 (15)	0.3040 (2)	0.7592 (3)	0.0386 (16)	
O9	0.7442 (12)	0.3724 (2)	0.6740 (3)	0.0312 (15)	
O10	1.0815 (13)	0.3740 (2)	0.5963 (3)	0.0339 (15)	
O11	1.2235 (14)	0.2826 (2)	0.5513 (3)	0.0365 (16)	
O12	0.7963 (14)	0.2174 (2)	0.6350 (3)	0.0361 (16)	

### Atomic displacement parameters (Å^2^)

**Table:**

	*U*^11^	*U*^22^	*U*^33^	*U*^12^	*U*^13^	*U*^23^
C1	0.031 (5)	0.030 (5)	0.037 (5)	0.006 (4)	0.004 (4)	−0.002 (4)
C2	0.031 (5)	0.034 (5)	0.043 (5)	0.006 (4)	0.007 (4)	0.001 (4)
C3	0.038 (5)	0.028 (5)	0.040 (5)	−0.007 (4)	0.006 (4)	−0.001 (4)
C4	0.027 (4)	0.027 (4)	0.035 (5)	−0.005 (3)	0.003 (4)	−0.002 (4)
C5	0.029 (5)	0.021 (4)	0.047 (6)	0.001 (3)	0.000 (4)	0.003 (4)
C6	0.032 (5)	0.024 (4)	0.042 (5)	−0.002 (4)	−0.002 (4)	0.002 (4)
C7	0.049 (6)	0.026 (5)	0.045 (6)	0.003 (4)	−0.010 (5)	0.004 (4)
C8	0.047 (6)	0.021 (5)	0.059 (7)	0.007 (4)	−0.005 (5)	−0.006 (4)
C9	0.034 (5)	0.034 (5)	0.046 (6)	0.008 (4)	0.003 (4)	−0.007 (4)
C10	0.036 (5)	0.031 (5)	0.038 (5)	0.000 (4)	−0.005 (4)	−0.001 (4)
C11	0.040 (5)	0.020 (4)	0.037 (5)	0.001 (4)	0.005 (4)	−0.005 (4)
C12	0.057 (7)	0.024 (5)	0.042 (6)	−0.002 (4)	0.014 (5)	−0.007 (4)
C13	0.037 (5)	0.025 (4)	0.037 (5)	0.003 (4)	0.008 (4)	0.000 (4)
C14	0.026 (5)	0.036 (5)	0.036 (5)	0.000 (4)	0.003 (4)	−0.003 (4)
C15	0.022 (4)	0.030 (5)	0.031 (4)	−0.002 (3)	0.006 (3)	−0.002 (4)
C16	0.027 (4)	0.028 (4)	0.033 (5)	−0.002 (4)	0.003 (4)	0.003 (4)
C17	0.057 (6)	0.016 (4)	0.040 (5)	0.002 (4)	0.000 (5)	0.000 (4)
C18	0.029 (5)	0.032 (5)	0.043 (5)	0.010 (4)	0.001 (4)	−0.001 (4)
C19	0.034 (5)	0.022 (4)	0.039 (5)	0.007 (4)	0.005 (4)	−0.005 (4)
C20	0.028 (5)	0.031 (5)	0.034 (5)	0.001 (4)	−0.002 (4)	−0.001 (4)
O1	0.030 (3)	0.029 (3)	0.042 (4)	0.001 (3)	0.005 (3)	−0.005 (3)
O2	0.043 (4)	0.033 (4)	0.038 (4)	0.008 (3)	0.005 (3)	0.003 (3)
O3	0.026 (3)	0.040 (4)	0.039 (4)	−0.001 (3)	0.009 (3)	0.003 (3)
O4	0.041 (4)	0.031 (4)	0.043 (4)	0.003 (3)	0.012 (3)	0.003 (3)
O5	0.045 (4)	0.032 (4)	0.049 (4)	0.009 (3)	0.018 (3)	−0.013 (3)
O6	0.030 (3)	0.030 (3)	0.038 (4)	−0.007 (3)	0.006 (3)	−0.008 (3)
C21	0.043 (6)	0.032 (5)	0.049 (6)	0.003 (4)	0.013 (5)	0.004 (4)
C22	0.043 (5)	0.031 (5)	0.033 (5)	0.001 (4)	0.008 (4)	0.007 (4)
C23	0.023 (4)	0.027 (4)	0.039 (5)	−0.001 (3)	0.002 (4)	0.004 (4)
C24	0.036 (5)	0.030 (5)	0.029 (4)	−0.005 (4)	0.003 (4)	−0.001 (4)
C25	0.038 (5)	0.018 (4)	0.037 (5)	−0.005 (4)	−0.008 (4)	0.001 (3)
C26	0.028 (4)	0.030 (5)	0.036 (5)	−0.006 (4)	−0.005 (4)	−0.002 (4)
C27	0.013 (4)	0.042 (5)	0.042 (5)	−0.003 (3)	−0.002 (3)	0.000 (4)
C28	0.034 (5)	0.025 (4)	0.037 (5)	0.003 (4)	0.000 (4)	0.005 (4)
C29	0.023 (4)	0.033 (5)	0.036 (5)	−0.002 (4)	0.002 (3)	−0.001 (4)
C30	0.023 (4)	0.021 (4)	0.041 (5)	−0.006 (3)	−0.005 (4)	−0.002 (4)
C31	0.027 (5)	0.033 (5)	0.034 (5)	−0.011 (4)	0.006 (4)	−0.007 (4)
C32	0.026 (4)	0.038 (5)	0.037 (5)	0.002 (4)	0.013 (4)	−0.008 (4)
C33	0.027 (4)	0.024 (4)	0.045 (5)	−0.005 (3)	0.000 (4)	0.000 (4)
C34	0.028 (4)	0.027 (4)	0.048 (6)	0.000 (4)	−0.003 (4)	−0.007 (4)
C35	0.036 (5)	0.031 (5)	0.046 (6)	−0.004 (4)	0.000 (4)	0.006 (4)
C36	0.024 (4)	0.039 (5)	0.052 (6)	0.001 (4)	0.006 (4)	−0.007 (5)
C37	0.035 (5)	0.031 (5)	0.066 (7)	−0.003 (4)	−0.005 (5)	−0.012 (5)
C38	0.044 (6)	0.027 (5)	0.057 (7)	0.000 (4)	0.004 (5)	−0.004 (5)
C39	0.042 (6)	0.020 (4)	0.054 (6)	0.005 (4)	0.007 (5)	0.004 (4)
C40	0.038 (5)	0.016 (4)	0.057 (6)	0.013 (4)	0.005 (5)	0.002 (4)
O7	0.036 (3)	0.025 (3)	0.043 (4)	−0.006 (3)	0.003 (3)	0.000 (3)
O8	0.042 (4)	0.029 (3)	0.045 (4)	0.006 (3)	0.012 (3)	0.006 (3)
O9	0.031 (3)	0.028 (3)	0.034 (3)	0.000 (3)	−0.002 (3)	0.005 (3)
O10	0.031 (3)	0.034 (4)	0.037 (4)	0.006 (3)	0.004 (3)	0.003 (3)
O11	0.040 (4)	0.030 (3)	0.040 (4)	−0.005 (3)	0.014 (3)	−0.007 (3)
O12	0.045 (4)	0.018 (3)	0.045 (4)	−0.011 (3)	0.007 (3)	−0.003 (3)

### Geometric parameters (Å, °)

**Table:**

C1—O1	1.437 (12)	C21—O7	1.443 (13)
C1—C2	1.530 (14)	C21—C22	1.491 (15)
C1—H1A	0.9900	C21—H21A	0.9900
C1—H1B	0.9900	C21—H21B	0.9900
C2—O2	1.420 (12)	C22—O8	1.435 (12)
C2—H2A	0.9900	C22—H22A	0.9900
C2—H2B	0.9900	C22—H22B	0.9900
C3—O2	1.397 (12)	C23—O8	1.396 (11)
C3—C4	1.532 (13)	C23—C24	1.518 (13)
C3—H3A	0.9900	C23—H23A	0.9900
C3—H3B	0.9900	C23—H23B	0.9900
C4—O3	1.452 (12)	C24—O9	1.418 (11)
C4—H4A	0.9900	C24—H24A	0.9900
C4—H4B	0.9900	C24—H24B	0.9900
C5—O3	1.355 (11)	C25—O9	1.394 (11)
C5—C10	1.391 (15)	C25—C26	1.402 (13)
C5—C6	1.406 (14)	C25—C30	1.414 (14)
C6—C7	1.392 (14)	C26—C27	1.384 (14)
C6—H6	0.9500	C26—H26	0.9500
C7—C8	1.361 (16)	C27—C28	1.402 (13)
C7—H7	0.9500	C27—H27	0.9500
C8—C9	1.397 (16)	C28—C29	1.393 (13)
C8—H8	0.9500	C28—H28	0.9500
C9—C10	1.383 (14)	C29—C30	1.383 (13)
C9—H9	0.9500	C29—H29	0.9500
C10—O4	1.355 (12)	C30—O10	1.368 (11)
C11—O4	1.441 (12)	C31—O10	1.420 (12)
C11—C12	1.502 (14)	C31—C32	1.508 (14)
C11—H11A	0.9900	C31—H31A	0.9900
C11—H11B	0.9900	C31—H31B	0.9900
C12—O5	1.419 (12)	C32—O11	1.437 (12)
C12—H12A	0.9900	C32—H32A	0.9900
C12—H12B	0.9900	C32—H32B	0.9900
C13—O5	1.466 (11)	C33—O11	1.435 (12)
C13—C14	1.500 (13)	C33—C34	1.528 (12)
C13—H13A	0.9900	C33—H33A	0.9900
C13—H13B	0.9900	C33—H33B	0.9900
C14—O6	1.446 (11)	C34—O12	1.423 (13)
C14—H14A	0.9900	C34—H34A	0.9900
C14—H14B	0.9900	C34—H34B	0.9900
C15—O6	1.368 (11)	C35—O12	1.370 (12)
C15—C16	1.379 (12)	C35—C36	1.371 (15)
C15—C20	1.384 (13)	C35—C40	1.407 (15)
C16—C17	1.429 (14)	C36—C37	1.405 (15)
C16—H16	0.9500	C36—H36	0.9500
C17—C18	1.353 (15)	C37—C38	1.376 (17)
C17—H17	0.9500	C37—H37	0.9500
C18—C19	1.422 (14)	C38—C39	1.419 (16)
C18—H18	0.9500	C38—H38	0.9500
C19—C20	1.371 (13)	C39—C40	1.367 (15)
C19—H19	0.9500	C39—H39	0.9500
C20—O1	1.369 (11)	C40—O7	1.372 (12)
			
O1—C1—C2	107.9 (8)	O7—C21—C22	107.4 (9)
O1—C1—H1A	110.1	O7—C21—H21A	110.2
C2—C1—H1A	110.1	C22—C21—H21A	110.2
O1—C1—H1B	110.1	O7—C21—H21B	110.2
C2—C1—H1B	110.1	C22—C21—H21B	110.2
H1A—C1—H1B	108.4	H21A—C21—H21B	108.5
O2—C2—C1	112.4 (8)	O8—C22—C21	114.2 (9)
O2—C2—H2A	109.1	O8—C22—H22A	108.7
C1—C2—H2A	109.1	C21—C22—H22A	108.7
O2—C2—H2B	109.1	O8—C22—H22B	108.7
C1—C2—H2B	109.1	C21—C22—H22B	108.7
H2A—C2—H2B	107.9	H22A—C22—H22B	107.6
O2—C3—C4	107.3 (8)	O8—C23—C24	107.5 (7)
O2—C3—H3A	110.3	O8—C23—H23A	110.2
C4—C3—H3A	110.3	C24—C23—H23A	110.2
O2—C3—H3B	110.3	O8—C23—H23B	110.2
C4—C3—H3B	110.3	C24—C23—H23B	110.2
H3A—C3—H3B	108.5	H23A—C23—H23B	108.5
O3—C4—C3	105.9 (7)	O9—C24—C23	107.1 (8)
O3—C4—H4A	110.6	O9—C24—H24A	110.3
C3—C4—H4A	110.6	C23—C24—H24A	110.3
O3—C4—H4B	110.6	O9—C24—H24B	110.3
C3—C4—H4B	110.6	C23—C24—H24B	110.3
H4A—C4—H4B	108.7	H24A—C24—H24B	108.6
O3—C5—C10	114.6 (8)	O9—C25—C26	123.4 (9)
O3—C5—C6	124.5 (9)	O9—C25—C30	115.8 (8)
C10—C5—C6	120.9 (9)	C26—C25—C30	120.6 (8)
C7—C6—C5	118.3 (10)	C27—C26—C25	118.6 (9)
C7—C6—H6	120.8	C27—C26—H26	120.7
C5—C6—H6	120.8	C25—C26—H26	120.7
C8—C7—C6	120.9 (10)	C26—C27—C28	120.4 (9)
C8—C7—H7	119.6	C26—C27—H27	119.8
C6—C7—H7	119.6	C28—C27—H27	119.8
C7—C8—C9	120.8 (9)	C29—C28—C27	121.3 (9)
C7—C8—H8	119.6	C29—C28—H28	119.4
C9—C8—H8	119.6	C27—C28—H28	119.4
C10—C9—C8	119.8 (10)	C30—C29—C28	118.7 (9)
C10—C9—H9	120.1	C30—C29—H29	120.7
C8—C9—H9	120.1	C28—C29—H29	120.7
O4—C10—C9	126.2 (10)	O10—C30—C29	124.7 (9)
O4—C10—C5	114.5 (9)	O10—C30—C25	115.0 (8)
C9—C10—C5	119.3 (9)	C29—C30—C25	120.3 (8)
O4—C11—C12	107.3 (8)	O10—C31—C32	107.8 (8)
O4—C11—H11A	110.2	O10—C31—H31A	110.1
C12—C11—H11A	110.2	C32—C31—H31A	110.1
O4—C11—H11B	110.2	O10—C31—H31B	110.1
C12—C11—H11B	110.2	C32—C31—H31B	110.1
H11A—C11—H11B	108.5	H31A—C31—H31B	108.5
O5—C12—C11	114.6 (9)	O11—C32—C31	113.3 (8)
O5—C12—H12A	108.6	O11—C32—H32A	108.9
C11—C12—H12A	108.6	C31—C32—H32A	108.9
O5—C12—H12B	108.6	O11—C32—H32B	108.9
C11—C12—H12B	108.6	C31—C32—H32B	108.9
H12A—C12—H12B	107.6	H32A—C32—H32B	107.7
O5—C13—C14	105.0 (8)	O11—C33—C34	105.2 (8)
O5—C13—H13A	110.7	O11—C33—H33A	110.7
C14—C13—H13A	110.7	C34—C33—H33A	110.7
O5—C13—H13B	110.7	O11—C33—H33B	110.7
C14—C13—H13B	110.7	C34—C33—H33B	110.7
H13A—C13—H13B	108.8	H33A—C33—H33B	108.8
O6—C14—C13	104.3 (7)	O12—C34—C33	103.2 (7)
O6—C14—H14A	110.9	O12—C34—H34A	111.1
C13—C14—H14A	110.9	C33—C34—H34A	111.1
O6—C14—H14B	110.9	O12—C34—H34B	111.1
C13—C14—H14B	110.9	C33—C34—H34B	111.1
H14A—C14—H14B	108.9	H34A—C34—H34B	109.1
O6—C15—C16	123.4 (8)	O12—C35—C36	125.4 (10)
O6—C15—C20	116.1 (8)	O12—C35—C40	115.8 (9)
C16—C15—C20	120.5 (9)	C36—C35—C40	118.8 (9)
C15—C16—C17	119.2 (9)	C35—C36—C37	120.8 (11)
C15—C16—H16	120.4	C35—C36—H36	119.6
C17—C16—H16	120.4	C37—C36—H36	119.6
C18—C17—C16	120.1 (9)	C38—C37—C36	120.5 (10)
C18—C17—H17	119.9	C38—C37—H37	119.8
C16—C17—H17	119.9	C36—C37—H37	119.8
C17—C18—C19	119.9 (9)	C37—C38—C39	118.7 (10)
C17—C18—H18	120.1	C37—C38—H38	120.7
C19—C18—H18	120.1	C39—C38—H38	120.7
C20—C19—C18	119.9 (9)	C40—C39—C38	120.3 (10)
C20—C19—H19	120.1	C40—C39—H39	119.9
C18—C19—H19	120.1	C38—C39—H39	119.9
O1—C20—C19	124.8 (9)	C39—C40—O7	124.1 (10)
O1—C20—C15	114.7 (8)	C39—C40—C35	120.9 (10)
C19—C20—C15	120.4 (9)	O7—C40—C35	115.0 (9)
C20—O1—C1	118.2 (7)	C40—O7—C21	118.7 (8)
C3—O2—C2	113.4 (8)	C23—O8—C22	113.6 (7)
C5—O3—C4	117.8 (8)	C25—O9—C24	117.9 (7)
C10—O4—C11	117.5 (8)	C30—O10—C31	117.0 (8)
C12—O5—C13	112.4 (8)	C33—O11—C32	114.0 (7)
C15—O6—C14	119.0 (7)	C35—O12—C34	116.6 (7)
			
O1—C1—C2—O2	72.6 (10)	O7—C21—C22—O8	71.1 (11)
O2—C3—C4—O3	−170.3 (7)	O8—C23—C24—O9	−171.1 (7)
O3—C5—C6—C7	−179.0 (9)	O9—C25—C26—C27	−175.9 (8)
C10—C5—C6—C7	0.4 (14)	C30—C25—C26—C27	−1.1 (12)
C5—C6—C7—C8	−0.2 (15)	C25—C26—C27—C28	−1.0 (13)
C6—C7—C8—C9	0.1 (16)	C26—C27—C28—C29	1.3 (14)
C7—C8—C9—C10	−0.3 (16)	C27—C28—C29—C30	0.5 (14)
C8—C9—C10—O4	−178.2 (10)	C28—C29—C30—O10	178.4 (8)
C8—C9—C10—C5	0.5 (15)	C28—C29—C30—C25	−2.5 (13)
O3—C5—C10—O4	−2.2 (12)	O9—C25—C30—O10	−2.7 (11)
C6—C5—C10—O4	178.3 (9)	C26—C25—C30—O10	−178.0 (8)
O3—C5—C10—C9	178.9 (9)	O9—C25—C30—C29	178.1 (8)
C6—C5—C10—C9	−0.6 (14)	C26—C25—C30—C29	2.8 (12)
O4—C11—C12—O5	74.0 (11)	O10—C31—C32—O11	69.6 (10)
O5—C13—C14—O6	−172.4 (7)	O11—C33—C34—O12	−169.3 (7)
O6—C15—C16—C17	−179.3 (9)	O12—C35—C36—C37	−179.8 (10)
C20—C15—C16—C17	−0.4 (14)	C40—C35—C36—C37	−1.9 (15)
C15—C16—C17—C18	1.0 (14)	C35—C36—C37—C38	3.6 (16)
C16—C17—C18—C19	−0.2 (14)	C36—C37—C38—C39	−2.8 (16)
C17—C18—C19—C20	−1.3 (14)	C37—C38—C39—C40	0.4 (16)
C18—C19—C20—O1	178.4 (9)	C38—C39—C40—O7	179.9 (9)
C18—C19—C20—C15	2.0 (14)	C38—C39—C40—C35	1.3 (15)
O6—C15—C20—O1	1.1 (12)	O12—C35—C40—C39	177.5 (9)
C16—C15—C20—O1	−177.9 (8)	C36—C35—C40—C39	−0.6 (14)
O6—C15—C20—C19	177.9 (9)	O12—C35—C40—O7	−1.2 (12)
C16—C15—C20—C19	−1.1 (14)	C36—C35—C40—O7	−179.2 (9)
C19—C20—O1—C1	−3.7 (13)	C39—C40—O7—C21	−14.8 (14)
C15—C20—O1—C1	172.9 (8)	C35—C40—O7—C21	163.8 (9)
C2—C1—O1—C20	177.6 (8)	C22—C21—O7—C40	−177.2 (8)
C4—C3—O2—C2	179.7 (8)	C24—C23—O8—C22	−175.7 (8)
C1—C2—O2—C3	−86.0 (10)	C21—C22—O8—C23	−86.7 (11)
C10—C5—O3—C4	−170.4 (8)	C26—C25—O9—C24	11.6 (12)
C6—C5—O3—C4	9.0 (13)	C30—C25—O9—C24	−163.5 (8)
C3—C4—O3—C5	175.2 (8)	C23—C24—O9—C25	168.2 (7)
C9—C10—O4—C11	−14.9 (14)	C29—C30—O10—C31	−10.2 (12)
C5—C10—O4—C11	166.3 (8)	C25—C30—O10—C31	170.6 (7)
C12—C11—O4—C10	−175.8 (8)	C32—C31—O10—C30	−174.1 (7)
C11—C12—O5—C13	−88.0 (11)	C34—C33—O11—C32	−179.3 (7)
C14—C13—O5—C12	−175.2 (8)	C31—C32—O11—C33	−86.3 (10)
C16—C15—O6—C14	17.5 (13)	C36—C35—O12—C34	6.7 (14)
C20—C15—O6—C14	−161.4 (8)	C40—C35—O12—C34	−171.2 (8)
C13—C14—O6—C15	166.0 (8)	C33—C34—O12—C35	−179.7 (8)

### Hydrogen-bond geometry (Å, °)

**Table:**

*D*—H···*A*	*D*—H	H···*A*	*D*···*A*	*D*—H···*A*
C16—H16···O8^i^	0.95	2.57	3.46 (1)	157
C26—H26···O5^ii^	0.95	2.54	3.42 (1)	155

Symmetry codes: (i) *x*+1/2, −*y*+1/2, *z*−1/2; (ii) *x*−1/2, −*y*+1/2, *z*+1/2.
